# Gestational VPA exposure reduces the density of juxtapositions between TH+ axons and calretinin or calbindin expressing cells in the ventrobasal forebrain of neonatal mice

**DOI:** 10.3389/fnana.2024.1426042

**Published:** 2024-07-04

**Authors:** Cintia Klaudia Finszter, Róbert Kemecsei, Gergely Zachar, Ágota Ádám, András Csillag

**Affiliations:** Department of Anatomy, Histology and Embryology, Faculty of Medicine, Semmelweis University, Budapest, Hungary

**Keywords:** nucleus accumbens, olfactory tubercle, ventral tegmental area, substantia nigra, mesolimbic pathway, calcium binding proteins, axon guidance, pattern formation

## Abstract

Gestational exposure to valproic acid (VPA) is a valid rodent model of human autism spectrum disorder (ASD). VPA treatment is known to bring about specific behavioral deficits of sociability, matching similar alterations in human autism. Previous quantitative morphometric studies from our laboratory showed a marked reduction and defasciculation of the mesotelencephalic dopaminergic pathway of VPA treated mice, along with a decrease in tissue dopamine in the nucleus accumbens (NAc), but not in the caudatoputamen (CPu). In the present study, the correlative distribution of tyrosine hydroxylase positive (TH+) putative axon terminals, presynaptic to the target neurons containing calretinin (CR) or calbindin (CB), was assessed using double fluorescent immunocytochemistry and confocal laser microscopy in two dopamine recipient forebrain regions, NAc and olfactory tubercle (OT) of neonatal mice (mothers injected with VPA on ED13.5, pups investigated on PD7). Representative image stacks were volumetrically analyzed for spatial proximity and abundance of presynaptic (TH+) and postsynaptic (CR+, CB+) structures with the help of an Imaris (Bitplane) software. In VPA mice, TH/CR juxtapositions were reduced in the NAc, whereas the TH/CB juxtapositions were impoverished in OT. Volume ratios of CR+ and CB+ elements remained unchanged in NAc, whereas that of CB+ was markedly reduced in OT; here the abundance of TH+ axons was also diminished. CR and CB were found to partially colocalize with TH in the VTA and SN. In VPA exposed mice, the abundance of CR+ (but not CB+) perikarya increased both in VTA and SN, however, this upregulation was not mirrored by an increase of the number of CR+/TH+ double labeled cells. The observed reduction of total CB (but not of CB+ perikarya) in the OT of VPA exposed animals signifies a diminished probability of synaptic contacts with afferent TH+ axons, presumably by reducing the available synaptic surface. Altered dopaminergic input to ventrobasal forebrain targets during late embryonic development will likely perturb the development and consolidation of neural and synaptic architecture, resulting in lasting changes of the neuronal patterning (detected here as reduced synaptic input to dopaminoceptive interneurons) in ventrobasal forebrain regions specifically involved in motivation and reward.

## Introduction

Administration of valproic acid (VPA) to laboratory rodents at a critical time window of gestation has been used for some time as an experimental model for the investigation of typical failure symptoms associated with autism spectrum disorder (ASD). A commonly used potent antiepileptic, anticonvulsant, and mood stabilizer (Cipriani et al., 2013), prenatal treatment with VPA is known to cause characteristic deficits of social behavior in the offspring *post-partum* (Rodier et al., 1996; Schneider and Przewlocki, 2005; Wagner et al., 2006; Kim et al., 2011; Moldrich et al., 2013; Roullet et al., 2013; for comprehensive reviews see Roullet et al., 2013; Nicolini and Fahnestock, 2018; Fereshetyan et al., 2021). VPA-treated animals also exhibit anxiety, depression-like behavior, and abnormal nociception thresholds (Wu et al., 2017; Mahmood et al., 2018). The validity of embryonic exposure to VPA as an ASD model has been extended also to a wide variety of nonmammalian species such as fish (Baronio et al., 2017; Chen et al., 2018) or birds (Nishigori et al., 2013; Sgadò et al., 2018; Lorenzi et al., 2019; Zachar et al., 2019; Adiletta et al., 2022). Of the putative pathogenetic/risk factors, the VPA challenge corresponds to the contribution of epigenetic/environmental factors in the pathogenesis of ASD (Ergaz et al., 2016), as opposed to proven, if rare, genetic components (Scherer and Dawson, 2011; Abrahams et al., 2013), or to other candidate genes, susceptibility factors, and signaling pathways, of diagnostic or predictive value (Satterstrom et al., 2020).

The principal mechanism through which VPA treatment evokes autism-like symptoms is likely an inhibition of histone deacetylase, modifying transcription (Göttlicher et al., 2001; Kataoka et al., 2013; Moldrich et al., 2013) at a critical time point of embryonic development. As evidenced by previous studies, E12-15 is the optimal time window of VPA administration for eliciting ASD-like behaviors in rats or mice (Kataoka et al., 2013) without major teratogenic alterations. This prenatal period coincides with the early differentiation of midbrain dopaminergic (DAergic) neurons (Bayer et al., 1995; Blaess et al., 2011) and with the initial outgrowth and migration of DAergic axons targeting the ventrobasal forebrain (Blakely et al., 2011).

By comparison to numerous behavioral studies, the reports on the anatomical correlates of gestational VPA treatment are rather scarce. Previous studies from our laboratory have directed attention to specific alterations of the mesotelencephalic (MT) DAergic pathway following VPA exposure. In 7-day-old postnatal (P7) mouse pups born to mothers injected with VPA on gestational day 13.5, we demonstrated a prominent defasciculation of the MT tract of VPA treated mice as compared to controls, based on whole-mount fluorescence immunostaining against tyrosine hydroxylase (TH), and the tissue clearing method iDISCO (Ádám et al., 2020). In the same study, a decrease of tissue DA in the nucleus accumbens, but not in the caudate-putamen (CPu), was also confirmed (Ádám et al., 2020). These findings have suggested that the reduction of DA, through perturbed growth and axon misguidance of the DAergic pathway (leading to defasciculation), observed in VPA-exposed animals at P7 (Ádám et al., 2020) should also interfere with ongoing pattern formation in the dopaminoceptive target regions of the mesotelencephalic pathway (particularly its mesoaccumbens/mesolimbic portion). Accordingly, we went on to investigate VPA-evoked changes at the level of cells (apoptosis-frequency, abundance, and distribution of calcium binding proteins (CBP)), as well as of synapses (general vs DAergic) in selected dopaminoceptive subpallial regions, as compared to pallial regions, at P7, the age at which the MT pathway reduction had been observed (Ádám et al., 2020). Enhanced apoptosis frequency on VPA was found in nearly all investigated subpallial and pallial regions, while calretinin (CR) expression was decreased in pallial (cortical) regions but not in the subpallium (Finszter et al., 2023). In the same study, calbindin (CB) was selectively reduced in the CPu of VPA exposed animals at P7 but no longer at P60, whereas TH protein was found to drop, without simultaneous decrease of the synaptic protein, synaptophysin in nucleus accumbens (NAc) (but not in CPu), pointing to a selective loss of TH+ synapses (Finszter et al., 2023).

We posited that the observed reduction in DAergic input to forebrain target regions (primarily NAc), at the critical time window of development, might perturb the shaping of neural and synaptic architecture in these regions, specifically involved in motivation and reward. The ventrobasal forebrain, including the NAc, receives dopaminergic input mainly from the ventral tegmental area (VTA), whereas the dorsolateral striatum or CPu receives input mainly from the substantia nigra, pars compacta (SNc) (*cf.* Hasue and Shammah-Lagnado, 2002; Rodríguez-López et al., 2017). Due to its composition and to the extensive connections with limbic forebrain centers (ventral pallidum (VP), extended amygdala, septum (sept), bed nucleus of stria terminalis (BNST), olfactory tubercle (OT), prefrontal cortex, and certain hypothalamic nuclei) the ventrobasal forebrain occupies a central position in decision-making based on learned associations, reward, and social attraction (Humphries and Prescott, 2010). Many of these functions are potential targets for impairment due to ASD. The mesolimbic dopaminergic reward system is amply interconnected and overlapping with the social brain network (Goodson, 2005) forming the phylogenetically conservative social decision-making network (O’Connell and Hofmann, 2011, 2012; Csillag et al., 2022).

The association of ASD with functional impairment (Hamilton et al., 2013), genomic alteration (Staal et al., 2012; Nguyen et al., 2014), or defective development (Bissonette and Roesch, 2016; Alsanie et al., 2017) of the DAergic system, has been noted for some time. Striatal circuits, receiving ample DAergic input, have been implicated in the development of various forms of ASD (for review see Fuccillo, 2016). DAergic dysfunction was also verified in animal models of autism (Chao et al., 2020; Kuo and Liu, 2022.). By now, these results seem to have culminated in a ‘dopamine theory’ of human autism (Hara et al., 2015; Pavăl, 2017; Pavăl and Micluţia, 2021; László et al., 2022). However, unlike most protagonists of this promising hypothesis, we prefer to emphasize the importance of DA as an early promoter of forebrain organization, as envisaged by Voorn et al. (1988) for striatal development, and evidenced, in *ex vivo* target neurons, by co-culture studies (Snyder-Keller et al., 2008). By extrapolation, the extensive pattern formation in the ventrobasal forebrain, constituting part of the social brain network, is likely being regulated by DAergic input. According to our working hypothesis, a weak or retarded DAergic input (*by* ‘missing’ the critical time point of development), may lead to inadequate adaptation to social signals, ultimately manifested as different forms of ASD.

The main objective of the current study was to further investigate the neuroanatomical sequels of gestational VPA exposure, in particular, those representing alterations to the pattern of dopaminergic innervation, in two specific dopamine receptive target regions of the ventrobasal forebrain, nucleus accumbens and tuberculum olfactorium, at P7. We opted for a quantitative morphometric analysis of close appositions (termed here juxtapositions) between TH+ axons and the perikarya or dendrites of CR+ or CB+ neurons.

The age of mice (P7) was chosen for comparability with our previous observations (Ádám et al., 2020; Finszter et al., 2023). The involvement of CBPs in VPA-related (Lauber et al., 2016), or 6-OHDA-evoked (Petryszyn et al., 2021) responses has been confirmed in striatal regions. Both CBPs under investigation have been shown to be present in the NAc, with a proven pattern of distribution, and neither CR+ nor CB+ cells showed quantitative changes in VPA treated animals at P7 (Finszter et al., 2023). Given a well-established and functionally stable background of these dopamine recipient neuronal populations, any differences in the abundance of TH/CR or TH/CB juxtapositions measured in VPA exposed, as compared to control, mouse pups may well serve as a model for meaningful changes in synaptic pattern formation of DAergic afferents.

Although no previous data on VPA-associated morphological changes of CBPs have been reported for the OT, it seemed well justified to extend our investigation also to this eminent limbic forebrain center (especially in a macrosmatic species).

## Materials and methods

### Experimental animals

For VPA treatment, 20 adult female C57BL mice (obtained from Janvier Labs, France) were used. The animals were mated by placing two females into a box in which a resident male had been kept for 24h before mating. The males were removed after 12 h of cohabitation. The progress of pregnancy was monitored by regular weighing of the animals, and only those with a steady gain of weight were considered pregnant and processed further (altogether 12 animals). The pregnant females were kept with *ad libitum* access to standard laboratory chow and water, under a 12/12 h light/dark cycle. On day 13.5 of pregnancy, one half of the mothers (6 animals) received intraperitoneal injection of 500 mg/kg body weight VPA (diluted at 100 mg/ml with physiological saline). The other half of pregnant mice (6 animals) were injected with vehicle as control animals, under identical conditions. After birth, the pups remained with the dams until day 7. A maximum of two littermates were used in each experimental group.

### Tissue preparation

Seven-day-old (P7) VPA-treated and control mice (2 female and 3 male pups per treatment, altogether 10 individuals), were terminally anesthetized with an intraperitoneal injection of ketamine (50 mg/ml) and xylazine (20 mg/ml) mixture of 2:1, 0.1 ml total volume. The animals were transcardially perfused with a solution of 4 % paraformaldehyde in PBS, at 4°C. After removal, the brains were postfixed in the same solution for at least 48 h.

### Immunocytochemistry

The brains were immersed in 30 % sucrose (until they sank to the bottom of the vial) and cut into 30 μm coronal sections with a Leica SM2000R freezing microtome. The sections were stored in 0.01 % of azide-containing PBS at 4°C until further processing. Six series of sections were prepared from each brain. We consistently applied the same technical procedure for the sections from control and those from VPA-treated animals, the specimens were handled simultaneously, using the same batch of reagents under the same conditions. The sections were washed for 3 x 10 min, in PBS, and blocked for 30 min in normal horse serum (NHS), 5 % in PBS. Then the sections were incubated with the respective combination of primary antisera (diluted in PBS with 1 % NHS and 0.3 % Triton-X-100), overnight.

The following primary antibodies were used: a-tyrosine hydroxylase 1:1000 rabbit (LOT 3870479, Merck Millipore); a-calretinin 1:1000 chicken (LOT 22024, Thermo Fisher Scientific); a-calbindin 1:1000 chicken (LOT 22654, Thermo Fisher Scientific). Following washing for 3 x 10 min, in PBS, the sections were incubated with the respective secondary antibodies: Donkey a-rabbit-Alexa-488, 1:1000 (LOT VC296619; Invitrogen); Goat a-chicken-Alexa-594, 1:1000 (LOT WA316328, Invitrogen) for 1 h, followed by another washing for 3 x 10 min, in PBS. The sections were mounted on glass slides and coverslipped using a glycerol/PBS mixture (1:1).

### Microscopy and cell counting

#### Abundance of immunoreactive cell bodies

For the counting of perikarya, the specimens were viewed and photographed under a Zeiss Jena, LSM 780 confocal laser microscope. On tile scans of 10x magnification, the reference frames were marked out according to the coordinates of the atlas by Paxinos and Franklin (2001), all labeled cells (in case of double labeling, separately for each fluorochrome) were counted manually, in representative coronal sections of each brain, following manual thresholding for cell body size. The latter meant that the diameter of every labeled cell to be counted was measured using the image viewer program Aperio ImageScope. Only those cell bodies which were larger than 5 μm in longest diameter were counted. Thresholding, counting, and further analysis were done on photographic images off-line, the images were coded by one observer and counted blind by another colleague, who was not aware of the code until the end of the analysis. The values were expressed as normalized density of cells per mm^2^.

Concerning the sampling procedure, single measuring frames were superimposed to representative coronal sections adapted from The Allen Mouse Brain Atlas (©2021 Allen Institute for Brain Science. Mouse Brain Connectivity),^1^ as defined consistently for each region of interest (ROI), at identical A/P levels in every brain.

**FIGURE 1:**
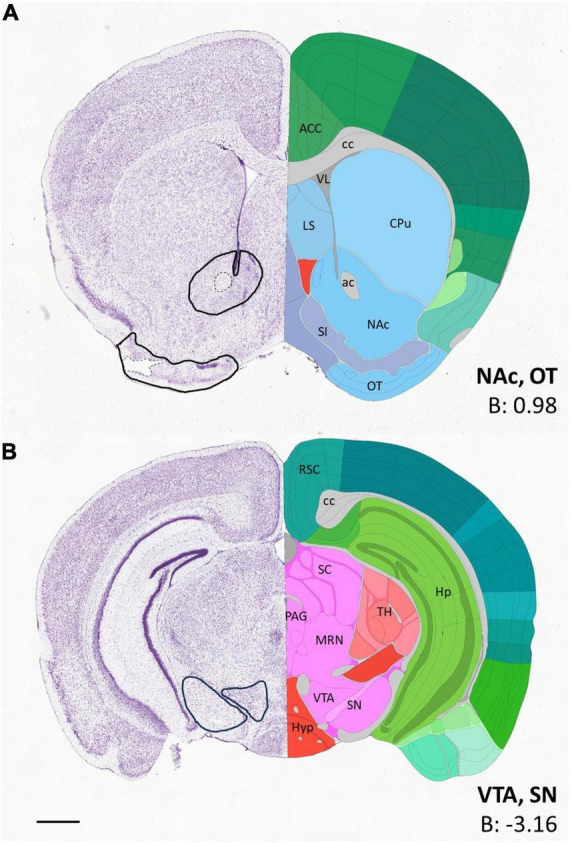
Diagrams depicting the location of sampling frames for each region of interest. **(A)** Shows the location of sampling frames for volumetric image analysis (using Imaris) in the nucleus accumbens and olfactory tubercle. **(B)** Shows the location of the sampling frames for cell counting in the ventral tegmental area and substantia nigra. The coronal images were adapted from Allen Brain Atlas (https://atlas.brain-map.org/), and marked according to Paxinos and Franklin (2001). The outlines of the measuring frames are projected onto the corresponding atlas image (left side), with some key landmarks (right side) and AP coordinates from Bregma (right corner). ac, anterior commissure; ACC, anterior cingulate cortex; B, Bregma; cc, corpus callosum; CPu, caudate-putamen; Hp, hippocampus; Hyp, hypothalamus; LS, lateral septum; MRN, midbrain reticular nucleus; NAc, nucleus accumbens; OT, olfactory tubercle; PAG, periaqueductal gray; RSC, retrosplenial cortex; SI, substantia innominata; SN, substantia nigra; TH, thalamus; VL, lateral ventricle; VTA, ventral tegmental area. Scale bar: 1 mm.

Both hemispheres were used for counting, but the values were not combined, since, in some cases, only one hemisphere remained intact. The measuring frames for each ROI were marked out by hand, to better fit to the boundaries of nuclei, to avoid noise from artifacts and to exclude non-relevant areas, such as brain pathways (e.g., the anterior commissure, in the case of NAc). The relevant anatomical landmarks and stereotaxic coordinates defining the sectional level, as well as the boundaries for each ROI, are summarized by diagrammatic drawings depicting the location of the measuring frames for every brain region analyzed (Figure 1).

Using the above method, we quantified the number of CB+, CR+ and TH+ cells in the VTA, SN, as well as that of CB+ and TH+ cells in the OT.

#### Volume density and spatial proximity of immunoreactive neuronal elements

For high-resolution quantitative microscopic analysis, one group of the mounted specimens were viewed and photographed under a LSM 780 confocal laser microscope (Zeiss Jena, Germany), the images were stitched using the ZEN 2010 program. Further analysis was carried out on Z-stacks of consistent total thickness (for each immunolabel and brain region), with a constant distance of 2 μm between single optical sections. Rather than applying systematic random sampling, we identified and picked a coronal section from each brain that contained the same required brain ROI and used this section as a representative of the whole region. To obtain the density values (normalized to area or volume), we standardized the denominator of the density measurement by systematically assigning a standard size ROI at a standard position (in relation to visible morphological landmarks) within the region. Anatomical precision and consistency of location took preference over comprehensiveness, especially since there are known subregional differences e.g., between the layers of OT, which, by random sampling, might increase the noise of measurement.

The confocal image stacks were analyzed using an Imaris software package (Bitplane AG, Switzerland, version 9.9.1.) operating on a HP Z4 control system.

To analyze the percentage of specific surface contacts between TH+ axons (presynaptic element) and CR+ or CB+ perikarya and their dendrites (postsynaptic elements), we followed previously elaborated guidelines of methodology (Schätzle et al., 2012; Fogarty et al., 2013; Klenowski et al., 2015). Briefly, confocal image stacks were loaded onto Imaris, and the immunoreactive fluorescent tissue elements were reconstructed in 3D with the help of the surface rendering function, for calculation of volumetric density of juxtaposed elements within the ROI. A representative example of the procedure is demonstrated by a short video (Supplementary Video 1), made from a selected image slab of NAc doubly stained against TH and CR, prior to quantitative analysis. Following manual rotation of the view angle, the TH+ axonal varicosity marked Juxt+ remains tightly apposed to a CR+ perikaryon, whereas the other TH+ bouton marked Juxt- appears to lose contact with the cell body, showing parallactic displacement of apparent position. The automated functions of Imaris are based essentially on such a procedure for selection of ‘true’ juxtapositions within the entire volume of ROI, without the need for the intervention of human operator.

**FIGURE 2:**
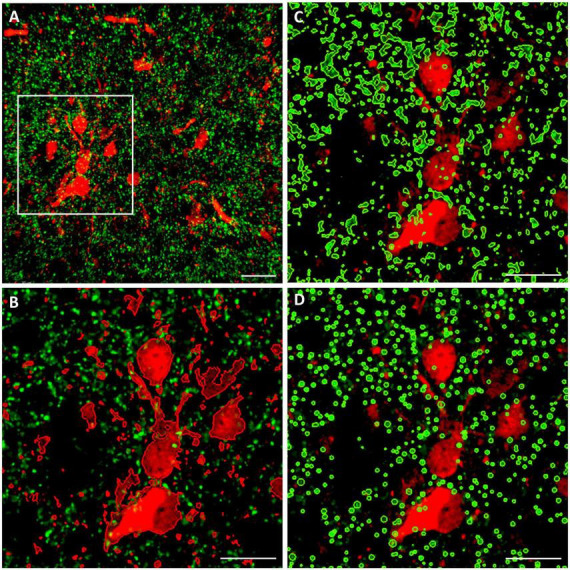
Representative confocal laser scanning images (top view of image stacks) demonstrating the methods for the quantitative analysis of close appositions (juxtapositions) between calbindin- immuoreactive (CB+) (red fluorochrome) and tyrosine hydroxylase immunoreactive (TH+) (green fluorochrome) tissue elements in the OT. Similar procedures were used for analysis of CB+/TH+ or calretinin immunoreactive (CR+) /TH+ juxtapositions in the NAc. **(A)** Source image stack loaded onto Imaris 9.9.1., with overlaid measuring frame of ROI; **(B)** surface rendering of red elements juxtaposed to green elements within the ROI; **(C)** surface rendering of green elements juxtaposed to red elements within the ROI; **(D)** particle rendering (with the help of spot detection function of Imaris) of green elements, appearing here as puncta juxtaposed to red elements (generated by surface rendering). For further details of volumetric analysis see section “Materials and methods.” Scale bar: 30 μm.

Two different volumetric approaches were applied for the quantitative analysis of juxtapositions, as demonstrated in Figure 2. The first one is based on spatial proximity of the surfaces enwrapping TH+ structural elements on the one hand, and those enveloping CR+ or CB+ structural elements on the other hand. For systematic surface renderings of tissue elements, the fluorescent level threshold was set to a fixed value (11 %), predetermined according to an optimal discernibility of the fluorescent signal, which was then used throughout the measurements. For determination of juxtapositions, the contact surface between the TH+ axons (green channel) and the CR+ or CB+ perikarya (red channel) was defined as the region with 0 μm separation between the rendered surfaces. The volume represented by the contacting surfaces as percent of total volume was measured by automated 3D computation. The consistency of automated computation was tested by rendering, in an image stack from OT (Figure 2A), the CB+ structures (red channel) juxtaposed to TH+ structures (green channel) (Figure 2B) or vice versa: TH+ structures juxtaposed to CB+ structures (Figure 2C). The resulting juxtaposition values did not differ from each other, indicating that the image analysis software reliably spotted the contacts between the structures labeled by the different fluorochromes, regardless of which one served as a reference.

The other method utilized the ‘spot detection’ function of Imaris, applied for the presynaptic (TH+) elements (Figure 2D). Since the axons and axonal boutons fall within a well-defined size range, it seemed reasonable to let Imaris generate puncta with a diameter of 1 μm and above, and a fluorescence intensity threshold of 5.5 %. Thereafter, the program counted those spots (generated from TH+ axons according to preset size and intensity parameters), that were located within a given distance (1 or 0 μm) from the surfaces rendered through 3D scanning of CB+ or CR+ structural elements. In our experience, albeit both two different methods yielded essentially similar results, the first approach (based upon bilateral surface rendering) resulted more often in tighter datasets with statistically significant differences between the treatment groups.

For assessment of the co-expression of labels within the same anatomical structure (TH and CB or TH and CR, in the TH+ axons) we applied the ‘Coloc’ feature of Imaris. This implements the Costes & Lockett method (Costes et al., 2004), based on automated selection of colocalized voxels, thereby eliminating operator-related bias. Colocalization of TH with CR or TH with CB was determined on a per-pixel basis. A summary of the above procedural steps is given in the workflow chart of Supplementary Figure 1.

#### Statistics

Statistical analysis of results was based on individual hemispheric data (with the animals as random factor), using the R Studio program. To determine the effects of treatment (VPA *vs* control) as fixed effects, we applied a generalized linear mixed model (GLMM) based on negative binomial regression with a Log link function (Yu et al., 2022). For analysis of the data of perikaryal counts in SN, VTA and OT, a negative binomial regression was used in every brain region. The linear mixed model included the data of the two hemispheres separately to control for within-subject variance but, since the ID of the individuals was included as a random effect, the two hemispheres’ data were NOT treated as statistically independent. Accordingly, the datapoints on the graphs represent the values of the given brain region from unilateral hemispheres, rather than the mean value of those regions from bilateral hemispheres. Notably, for the statistical analysis, the measured variables (spots, surface contacts) were taken into account, whilst the values of area/volume were used only as an offset variable.

## Results

### CB, CR, and TH perikarya in VTA and SN

**FIGURE 3:**
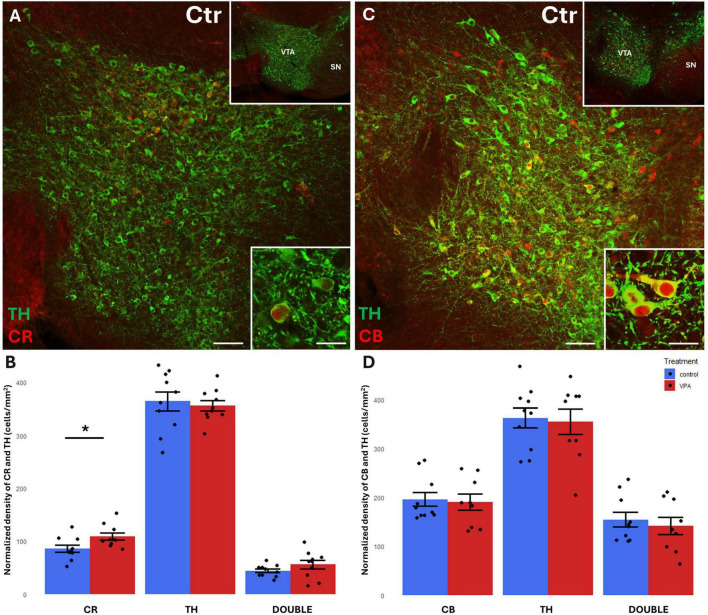
Representative confocal laser scanning images demonstrating the colocalization between tyrosine hydroxylase (TH) immunolabeled perikarya and those immunoreactive to calretinin **(A)** or calbindin **(C)** in the VTA of control mice (Scale bar: 50 μm). Lower magnification images of the regions are presented in the top right insets. Characteristic cell types of the region are shown under high magnification (Scale bar: 20 μm) in the bottom right insets. The diagrams on panels **(B,D)** show the areal density of CR+, TH+ and double labeled perikarya **(B)** as well as that of CB+, TH+ and double labeled perikarya **(D)** in VTA, comparing VPA-exposed and control P7 mice (*n* = 10 hemispheres, representing 5 animals per treatment group). The values are expressed as M ± S.E.M. *<0.05. CB, calbindin; CR, calretinin; Ctr, control; SN, substantia nigra; TH, tyrosine hydroxylase; VTA, ventral tegmental area.

**FIGURE 4:**
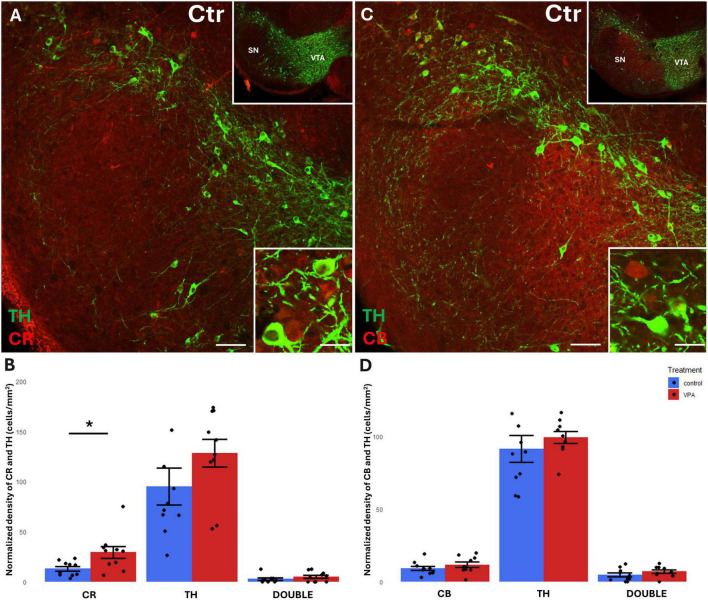
Representative confocal laser scanning images demonstrating the colocalization between tyrosine hydroxylase (TH) immunolabeled perikarya and those immunoreactive to calretinin **(A)** or calbindin **(C)** in the SN of control mice (Scale bar: 50 μm). Lower magnification images of the regions are presented in the top right insets. Characteristic cell types of the region are shown under high magnification (Scale bar: 20 μm) in the bottom right insets. The diagrams on panels **(B,D)** show the areal density of CR+, TH+ and double labeled perikarya **(B)** as well as that of CB+, TH+ and double labeled perikarya **(D)** in SN, comparing VPA-exposed and control P7 mice (*n* = 10 hemispheres, representing 5 animals per treatment group). The values are expressed as M ± S.E.M. *<0.05. Abbreviations as in Figure 3.

Before focusing on the ventrobasal limbic target regions, we first looked at the mesencephalic source regions (VTA, SN) of the MT for any differences in the abundance of CR+, CB+ and TH+ cell bodies between VPA-exposed and control mice. We found a significant increase of CR+, but not CB+, perikarya, in both regions, in the VPA-treated group (Figures 3, 4). No significant changes of TH+ cells were detected in either region (Figures 3, 4).

### Colocalization of CBPs with TH in the perikarya of mesencephalic source regions

A considerable percentage of TH+ cell bodies of both SN and VTA were observed to co-express CR or CB (Figures 3A, C, 4A, C). In the VTA, a substantial percentage (14–16%) of all TH+ neurons also contained CR (Figure 3B), and 40–42% of all TH+ neurons were double labeled for CB (Figure 3D). The density of CR+ neurons was significantly increased in the VPA exposed group (Figure 3B), however, the abundance of those cells double labeled for CR+/TH+ was not enhanced, nor did those cells double labeled against CB+/TH+ show a significant difference in number between the treatment groups (Figures 3B, D). In the SN, the percentage of CBP containing neurons was considerably less than in VTA; CR+ cells amounted to approx. 5–6% of all TH+ neurons, whereas CB+ cells constituted 7–9% of TH+ neurons (Figures 4B, D). Here again, the only significant change on VPA treatment was an increase of the density of CR+ neurons but, of the double labeled neurons, neither those positive for CR+/TH+, nor those positive for CB+/TH+ did show any significant difference between the treatment groups.

### Non-colocalization of CBPs with TH in the axons innervating NAc and OT

Neither of the CBPs under investigation were detected in substantial amount in the axons expressing TH, in the NAc or the in OT. As determined by the Coloc function of Imaris, with the widest detection window (meeting the strictest criteria for colocalized emittance), the colocalization ratios for CB/TH were: 0.38% (VPA), 0.39% (control) in OT; 0.39% (VPA), 0.36% (control) in NAc; whereas those for CR/TH were 0.29% (VPA), 0.26% (control) in NAc.

### TH/CB and TH/CR axosomatic/axodendritic juxtapositions in NAc

**FIGURE 5:**
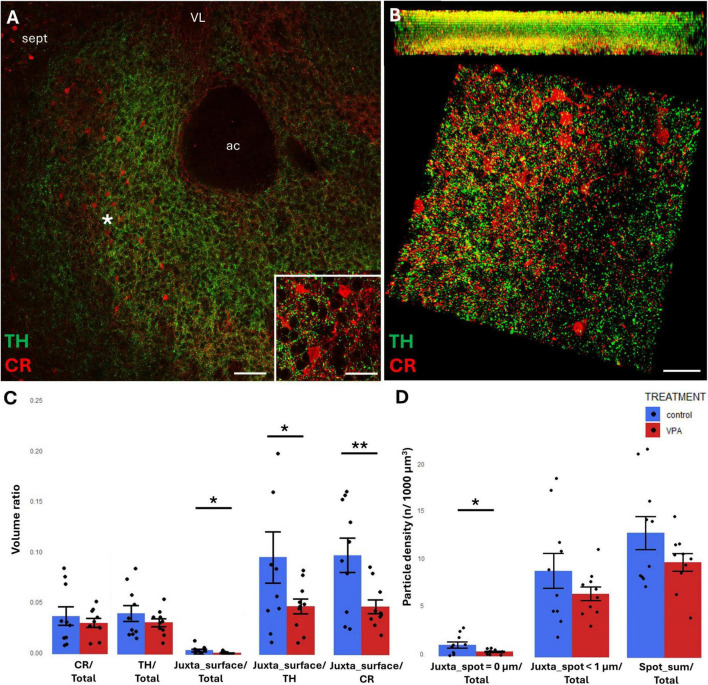
Representative confocal laser scanning images demonstrating the simultaneous occurrence of TH+ axons (presynaptic elements) together with CR+ **(A,B)** perikarya and dendrites (postsynaptic elements) in the NAc of P7 mice. **(A)** Overview image from control mice (Scale bar: 50 μm), characteristic cell types of the region are shown under higher magnification in insets (Scale bar: 20 μm). **(B)** High magnification quasi-3D reconstruction image from control mice rendered with the Imaris image analysis software (for location of sampling in the core of NAc see white asterisk in panel **(A)** The top panel demonstrates Z-stacks (side views exposing the Z axis), whereas the bottom panels show tilted image stacks viewed from the top, exposing the X-Y axes (Scale bar: 30 μm). **(C,D)** The diagrams show the results of quantitative volumetric analysis of juxtapositions in VPA-exposed animals (VPA, red columns) compared to control animals (control, blue columns). Two different methods were applied for computation. The first was based on the reconstruction of both the TH+ tissue elements (essentially axons) and the CR+ **(C)** elements (essentially perikarya and dendrites) using the surface rendering function, expressed as volume ratios. First, the total ratios of TH+ and CR+ structures were calculated (graphs denoted TH+/Total and CR+/Total, respectively). Then, the volume ratio of contacting surfaces (distance = 0 μm) of TH+ *vs.* CR+ structures was calculated using the respective function of Imaris. The resulting values were divided either by the total surface of ROI (graph denoted as Juxta_surface/Total), or alternatively, by the whole surface of TH+ (graph denoted Juxta_surface/TH) or CBP+ elements (graphs denoted Juxta_surface/CR). Using the other method (image panel **D**), the presynaptic elements (TH+ axons) were visualized as puncta generated by the spot detection function of Imaris. First, all puncta (particles) that passed the threshold of a diameter of 1 μm were counted by the program, and the values were divided by the total area of ROI (graph denoted Spot_sum/Total). Next, we measured the number of those spots which fell within a given distance (1 or 0 μm) to the rendered surface of CR+ **(D)** structural elements, divided by the total area of ROI (graphs denoted Juxta_spot < 1 μm/Total; Juxta_spot = 0 μm/Total, respectively), comparing P7 control and VPA-exposed mice (*n* = 10 hemispheres, representing 5 animals per treatment group). For further details see section “Materials and methods.” The values are expressed as M ± S.E.M. *<0.05, **<0.01. ac, anterior commissure; CR, calretinin; sept, septum; TH, tyrosine hydroxylase; VL, lateral ventricle.

**FIGURE 6:**
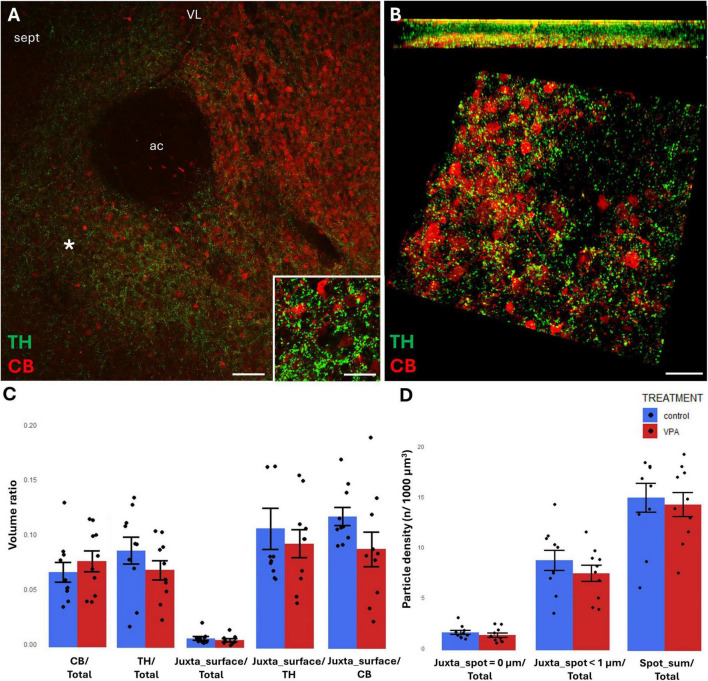
Representative confocal laser scanning images demonstrating the simultaneous occurrence of TH+ axons (presynaptic elements) together with CB+ **(A,B)** perikarya and dendrites (postsynaptic elements) in the NAc of P7 mice. **(A)** Overview image from control mice (scale bar: 50 μm), characteristic cell types of the region are shown under higher magnification in insets (scale bar: 20 μm). **(B)** High magnification quasi-3D reconstruction image from control mice rendered with the Imaris image analysis software (for location of sampling in the core of NAc see white asterisk in panel **(A)**. The top panel demonstrates Z-stacks (side views exposing the *Z* axis), whereas the bottom panels show tilted image stacks viewed from the top, exposing the X-Y axes (Scale bar: 30 μm). **(C,D)** The diagrams show the results of quantitative volumetric analysis of juxtapositions in VPA-exposed animals (VPA, red columns) compared to control animals (control, blue columns). Two different methods were applied for computation. The first was based on the reconstruction of both the TH+ tissue elements (essentially axons) and the CB+ **(C)** elements (essentially perikarya and dendrites) using the surface rendering function, expressed as volume ratios. First, the total ratios of TH+ and CB+ structures were calculated (graphs denoted TH+/Total and CB+/Total, respectively). Then, the volume ratio of contacting surfaces (distance = 0 μm) of TH+ *vs.* CB+ structures was calculated using the respective function of Imaris. The resulting values were divided either by the total surface of ROI (graph denoted as Juxta_surface/Total), or alternatively, by the whole surface of TH+ (graph denoted Juxta_surface/TH) or CBP+ elements (graphs denoted Juxta_surface/CB). Using the other method (image panel **D**), the presynaptic elements (TH+ axons) were visualized as puncta generated by the spot detection function of Imaris. First, all puncta (particles) that passed the threshold of a diameter of 1 μm were counted by the program, and the values were divided by the total area of ROI (graph denoted Spot_sum/Total). Next, we measured the number of those spots which fell within a given distance (1 or 0 μm) to the rendered surface of CB+ **(D)** structural elements, divided by the total area of ROI (graphs denoted Juxta_spot < 1 μm/Total; Juxta_spot = 0 μm/Total, respectively), comparing P7 control and VPA-exposed mice (*n* = 10 hemispheres, representing 5 animals per treatment group). For further details see section “Materials and methods.” The values are expressed as M ± S.E.M.; ac, anterior commissure; CB, calbindin; sept, septum; TH, tyrosine hydroxylase; VL, lateral ventricle.

In the NAc, the region of investigation encompassed the core (see the overview images from control mouse (Figures 5A, 6A), and the matching reconstruction images rendered with the help of Imaris image analysis software (Figures 5B, 6B). No change in volume ratio dependent on treatment (VPA exposed vs. control) was detected either with CR+ (Figure 5C) or CB+ (Figure 6C) structures. Using the same method, we also determined the volume ratio of TH+ elements. Here, the values were not significantly different in the control and VPA exposed groups (Figures 5C, 6C).

TH/CR juxtapositions were significantly reduced in VPA mice, both when measured with bilateral surface rendering, taking either TH or CR as reference (graphs Juxta_surface/TH and Juxta_surface/CR, respectively, of Figure 5C) and by spot detection of TH+ elements combined with surface rendering of CR+ elements (graph Juxta_spot = 0 μm of Figure 5D). Notably, although the tendency was similar, the latter difference failed to attain significance when the distance between the adjacent structural elements was extended to 1 μm (graph Juxta_spot < 1 μm of Figure 5D).

By contrast, TH/CB juxtapositions did not differ significantly between control and VPA exposed samples in NAc, regardless of the method applied (graphs Juxta_surface/TH, Juxta_surface/CB of Figure 6C, and Juxta_spot < 1 μm, Juxta_spot = 0 μm of Figure 6D).

### TH/CB axosomatic/axodendritic juxtapositions in OT

**FIGURE 7:**
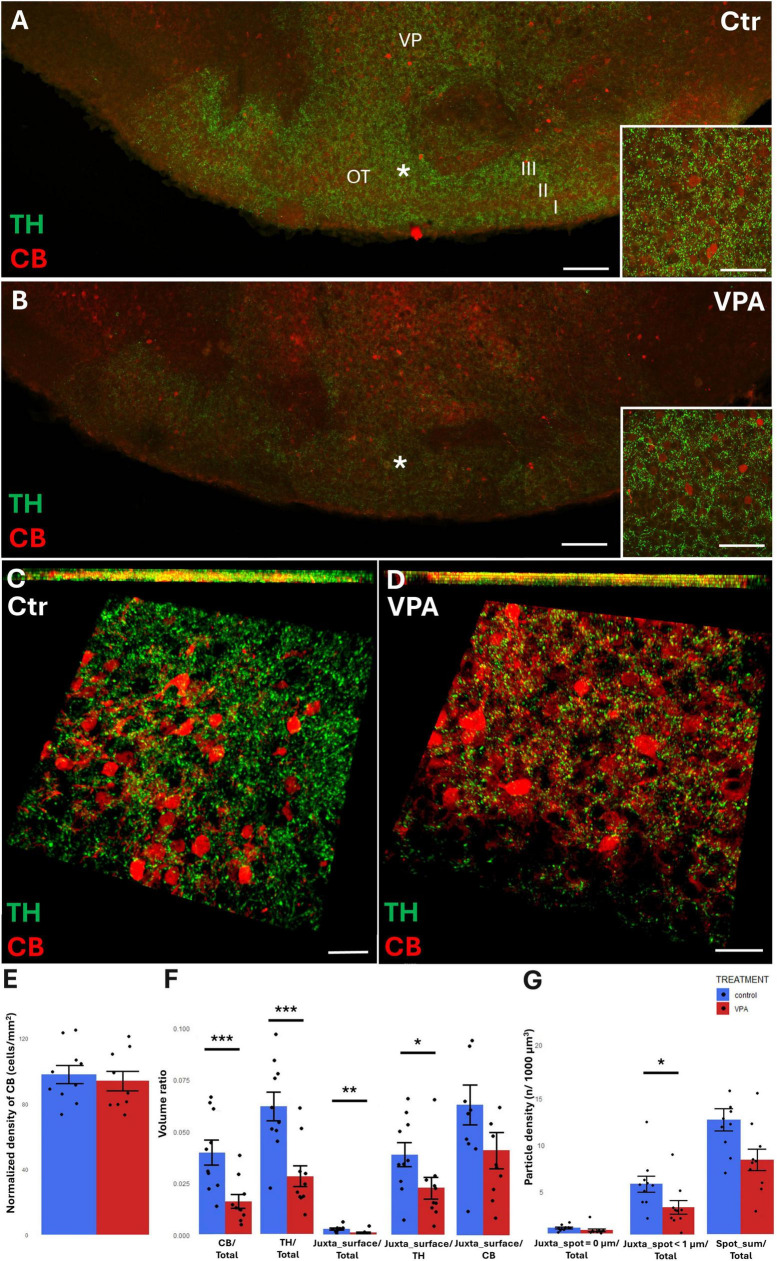
Representative confocal laser scanning images demonstrating the simultaneous occurrence of TH+ axons (presynaptic elements) together with CB+ perikarya and dendrites (postsynaptic elements) in the OT of P7 mice. **(A,B)** Overview images from control **(A)** and VPA-exposed **(B)** mice (scale bar: 50 μm), characteristic cell types of the region are shown under higher magnification in insets (scale bar: 20 μm). **(C,D)** High magnification quasi-3D reconstruction images from control **(C)** and VPA-exposed **(D)** mice rendered with the Imaris image analysis software (for location of sampling in layer III of OT see white asterisks in panels **(A,B)**. The top panels demonstrate Z-stacks (side views exposing the *Z* axis), whereas the bottom panels show tilted image stacks viewed from the top, exposing the X-Y axes (Scale bar: 30 μm). **(E)** The diagram shows the areal density of CB+ perikarya in OT, comparing VPA-exposed and control P7 mice (*n* = 10 hemispheres, representing 5 animals in both treatment groups). **(F,G)** The diagrams show the results of quantitative volumetric analysis of juxtapositions in VPA-exposed animals (VPA, red columns) compared to control animals (control, blue columns). Two different methods were applied for computation. The first approach (image panel **F**) was based on the reconstruction of both the TH+ tissue elements (essentially axons) and the CB+ elements (essentially perikarya and dendrites) using the surface rendering function, expressed as volume ratios. First, the total ratios of TH+ and CB+ structures were calculated (graphs denoted TH+/Total, and CB+/Total, respectively). Then, the volume ratios of contacting surfaces (distance = 0 μm) of TH+ *vs.* CB+ structures were calculated using the respective function of Imaris. The resulting values were divided either by the total surface of ROI (graph denoted as Juxta_surface/Total), or alternatively, by the whole surface of TH+ (graph denoted Juxta_surface/TH) or CB+ elements (graphs denoted Juxta_surface/CB). Using the other method (image panel **G**), the presynaptic elements (TH+ axons) were visualized as puncta generated by the spot detection function of Imaris. First, all puncta (particles) that passed the threshold of a diameter of 1 μm were counted by the program, and the values were divided by the total area of ROI (graph denoted Spot_sum/Total). Next, we measured the number of those spots which fell within a given distance (1 or 0 μm) to the rendered surface of CB+ structural elements, divided by the total area of ROI (graphs denoted Juxta_spot < 1 μm/Total; Juxta_spot = 0 μm/Total, respectively). For further details see section “Materials and methods.” The values are expressed as M ± S.E.M. *<0.05, **<0.01, ***<0.001. CB, calbindin; Ctr, control; TH, tyrosine hydroxylase; OT, olfactory tubercle (I-III layers); VP, ventral pallidum; VPA, valproate exposed.

In the OT, the region of investigation was set to layer III (see the overview images from control (Figure 7A) and VPA treated (Figure 7B) mice, and the matching reconstruction images rendered with the help of the Imaris image analysis software (Figures 7C, D). We determined the density of CB+ perikarya, and also measured the total volume ratio of CB by surface rendering. No change was detected in cell density (Figure 7E), whereas, by the surface rendering method, a significant decrease of CB was found in VPA-exposed animals, as compared to controls (graph CB/Total of Figure 7F). Abundance of TH+ elements (essentially axons) were also markedly reduced in the OT of VPA treated mice (graph TH/Total of Figure 7F). CR+ cells were only sporadically observed in the given ROI; therefore, quantitative analysis of these cells was not considered to be feasible.

By contrast with the situation in the NAc, TH/CB juxtapositions of the OT were significantly reduced in VPA exposed mice, both when measured with bilateral surface rendering, setting TH as reference (graph Juxta_surface/TH of Figure 7F), and by spot detection of TH+ elements combined with surface rendering of CR+ elements (graph Juxta_spot < 1 μm of Figure 7G).

## Discussion

### CB, CR, and TH perikarya in VTA and SN

We found a significant elevation of CR+, but not CB+, perikarya, in the VPA-treated group in both of dopaminergic midbrain regions. No significant changes of TH+ cells were detected in either region. However, the tendencies of changes seem to correspond to our earlier observation (decreased density of TH in VTA and elevated density of TH in SN; Ádám et al., 2020), which was based upon computerized reconstruction and cell counting in cleared whole-mount specimens (iDISCO method). Apparently, that technique permitted a more refined and complete analysis of entire brain regions than the currently performed cell counting method using spatially limited sampling. This likely explains why the present results regarding TH+ cells fail to reach the level of significance (even if the tendencies do not contradict to those published previously). The marked elevation of CR in the VTA and SN of VPA-exposed animals, as reported in the present study, may signify a response to the loss and derangement of the axons emanating from the source neurons, as shown by Ádám et al. (2020) for TH+ projections.

### Colocalization of CBPs with TH in the perikarya of mesencephalic source regions

In agreement with the literature data (Liang et al., 1996; Nemoto et al., 1999; Mongia et al., 2019), a substantial percentage of CBP containing neurons were double labeled, co-expressing TH, (altogether more in the VTA than in SN). Notably, only the CR+ cells showed an enhanced expression in the VPA treated group in both the VTA and SN. However, those cells which were double labeled for CR and TH did not show a significant difference in number between the treatment groups.

### Non-colocalization of CBPs with TH in the axons innervating NAc and OT

In the present study, we did not intend to further elaborate on the degree of perikaryal colocalizations, the problem has been extensively dealt with in other studies (Liang et al., 1996; Nemoto et al., 1999; Mongia et al., 2019). More importantly, however, we had to assess the degree of TH+ - CBP colocalization (if any) within the axons heading for the forebrain targets (NAc, OT). A substantial co-expression here would have presented a serious obstacle when evaluating TH/CBP juxtapositions within the target regions. Fortunately, neither of CBPs were detected in substantial amount in the axons expressing TH, in either the NAc or the OT. Since CB and CR are known to be present in many TH+ cell bodies of VTA, including those projecting on NAc and OT (Mongia et al., 2019), and CR was even found to upregulate in the VPA-exposed group (present study), the scarcity of CB/TH or CR/TH co-expression in the axons innervating the ventrobasal forebrain is somewhat unexpected. We assume that, in young postembryonic mice, the CBPs coexpressed in TH+ neurons remain close to the perikaryal compartment, rather than migrating along with the outgrowing axons, or at least they fail to enter the telencephalic target regions by P7. Of the sparse colocalizations still detected here, at least with CB/TH, some may correspond to a contingent of VGluT2-CB neurons of VTA, with proven projections to the NAc (Mongia et al., 2019).

### TH/CB and TH/CR axosomatic and axodendritic juxtapositions in NAc and OT

The key findings of the current study are as follows: In VPA mice, TH/CR juxtapositions are significantly reduced in the NAc core, while no similar change was observed in the case of TH/CB juxtapositions. However, the TH/CB juxtapositions were strongly reduced in layer III of the OT. The volume ratio of CR+ and CB+ elements remained unchanged in the NAc, whereas that of CB+ structures was markedly reduced in the OT.

Regarding the NAc, surface rendering of select immunoreactive structures, required for the determination of juxtapositions, enabled a re-evaluation of the abundance of CR+ and CB+ structures in relation to our previously reported observation (Finszter et al., 2023). Rather than counting CBP expressing cell bodies in representative fluorescent microscopic images (as reported previously), we now measured the total CB or CR content in confocal image stacks, expressed as the volume enclosed by the surface rendered for each marker as percent of total volume (volume ratio). This means that not only perikarya but also neuronal processes were taken into account. Notably, the present results did not differ from those of the previous report (Finszter et al., 2023), i.e., no change of CB or CR in VPA exposed vs. control P7 mice was detected, regardless of the measuring technique.

Since we did not specifically analyze the OT previously (Finszter et al., 2023), we now did both the counting of CB+ perikarya, and the measurement of total CB by surface rendering. Apparently, CR+ cells were too scarce in the OT, at least at this age, to be analyzed. Curiously, while no decrease in CB+ perikarya could be detected by ‘classical’ cell counting, the surface rendering approach yielded here a prominent drop of CB in the OT of VPA-exposed, compared to control, animals. This finding may indicate an overall reduction in the arborization of CB+ neurons within the OT (without a significant loss of perikarya), one possible reason for the observed reduction of TH+/CB+ juxtapositions.

Abundance of TH+ structures (essentially axons) was also reduced in the OT of VPA treated mice. This phenomenon has been noted previously as a qualitative observation (Finszter et al., 2023), now substantiated in a quantitative manner by the present report. On the other hand, the current observation on the abundance of TH+ elements in the NAc (Figures 5C, 6C) does not quite concur with (though does not directly contradict to) the previously reported decrease of DA in the NAc following VPA exposure (Ádám et al., 2020). The apparent controversy is possibly due to the fact that the brain samples of NAc dissected for the DA assay of the quoted study (Ádám et al., 2020) also contained parts of the OT (duly noted in Methods). Apparently, an intense response of OT far outweighing its mass ratio might have biased the overall result, then attributed to the NAc. Yet, despite the likely contribution of OT, the VPA-associated decrease of TH protein, determined by WB, could only be detected as a non-significant trend in the NAc (with no change in the CPu) in our previous study (Finszter et al., 2023). However, when normalized by synaptophysin, the drop of TH in VPA exposed animals became already prominent, indicating a strong reduction of TH+ axons in relation to the available synaptic sites. In other words, the likelihood of TH+ axons establishing synaptic contacts in the NAc (notably, the WB sample termed ‘NAc’ also included the OT) seems to have decreased. Our current findings on a significant reduction of TH+/CB+ juxtapositions in OT, and of TH+/CR+ juxtapositions in NAc seem to support this suggestion. Converging evidence suggests a diminished occurrence of those DAergic fibers establishing synaptic contacts, at least in two dopamine recipient regions of the ventrobasal forebrain, NAc and OT. Moreover, the results of the present study help specify a group of CBP expressing interneurons as potential targets for synaptic reorganization, following alteration of DAergic input to the ventrobasal forebrain, in VPA exposed mice.

Several studies have already pointed to the importance of synaptic reorganization in limbic forebrain regions, from enhanced dendritic arborization and spinogenesis in NAc, likely representing defective pruning (Bringas et al., 2013), to reduced spine density and lower expression of synaptic protein mRNAs in the hippocampus of VPA-exposed mice (Yamaguchi et al., 2017). Most of these observations were made on adult or adolescent (in any case, post-weaning) animals, i.e., after the consolidation of dopaminergic organization, starting in the late embryonic period, yet undergoing prolonged postnatal development (Antonopoulos et al., 2002). Our results from P7 mice attempt to tackle those processes which occur at the early phase of pattern formation, prior to cessation of the ingrowth and synapse formation of DA ergic fibers and their recipient structural elements.

The question rises to what extent can the observed changes of pattern formation and synaptic organization be ascribed to defective DA input to the limbic forebrain. There is little doubt that a definite defasciculation, affecting the TH+ fibers of the mesolimbic tract, is taking place in VPA exposed mouse pups, evident at P7 (Ádám et al., 2020). Similar alterations of the mesolimbic pathway have been reported to occur also in autistic children (Supekar et al., 2018). This may lead to a net loss of fibers reaching the limbic forebrain targets, or a misdirection of tegmental DAergic fibers from ventral (limbic) to dorsal striatum. We also reported a decrease of DA (measured by ELISA) in the NAc, but not in CPu (however, most of this effect may have been due to a disproportionately high contribution by OT) (Ádám et al., 2020). Both the decrease of the synaptic component of TH protein in ‘NAc’ (including OT) (as determined by immunoblotting) (Finszter et al., 2023) and the diminished incidence of TH+/CR+ or TH+/CB+ juxtapositions in NAc and OT, respectively, (current finding) seem to support the notion of a genuine attenuation of dopaminergic input at a critical time point of early postembryonic development. Whilst other independent literature sources did not confirm a reduction of TH+ cells in VTA, nor did they find a decrease in TH protein in the NAc at P21 (Maisterrena et al., 2022), there are also reports on the upregulation of DA receptors, in the NAc of rats born to VPA-exposed mothers (Schiavi et al., 2019), likely reflecting a compensatory mechanism following earlier deprivation of ligand (i.e., DA), as indeed it was detected by us in P7 mice. Selective depletion of TH in the ventrobasal forebrain regions, OT and NAc (shell), but not in the dorsal striatum, together with an upregulation of D1 and D2 receptors and phosphorylated DARPP-32 have been observed in En1^cre/+^, Otx2^flox/flox^ mutant mice, following a prominent reduction of midbrain DAergic neurons (Borgkvist et al., 2006). Apparently, the dopamine recipient ventrobasal forebrain is highly vulnerable to genomic and transcriptional defects affecting axonal pathfinding and neural organization. Based upon accumulating literature data (for review see Cansler et al., 2020), also supported by our current findings, pattern formation in the OT is likely to play a prominent role in early DAergic regulation.

The present results bear relevance also for an alternative/additional element of synaptic reorganization: alterations on the recipient side. The observed reduction of total CB (but not of CB+ perikarya) in the OT of VPA exposed animals (not reported previously) may further decrease the probability of synaptic contacts with afferent TH+ axons, presumably by reducing the available synaptic surface. Whether such reduction is due to enhanced pruning of dendritic spines, remains to be investigated. In any case, the declining ability of just one well-defined neuronal type (CB+) to receive dopaminergic input may exemplify and predict lasting synaptic reorganization, similar to those observed in non-human primates (Martin and Cork, 2014) in motivation-associated forebrain regions (such as OT), potentially leading to failure symptoms characteristic of human ASD.

## Data availability statement

The original contributions presented in this study are included in this article/Supplementary material, further inquiries can be directed to the corresponding author.

## Ethics statement

The animal study was approved by the Food Chain Safety and Animal Health Directorate of the Government Office for Pest County, Hungary (XIV-I-001-2269-4/2012). The study was conducted in accordance with the local legislation and institutional requirements.

## Author contributions

CF: Data curation, Investigation, Methodology, Software, Validation, Visualization, Writing – original draft. RK: Data curation, Methodology, Software, Validation, Writing – review and editing. GZ: Data curation, Funding acquisition, Methodology, Project administration, Resources, Software, Supervision, Validation, Writing – review and editing. ÁÁ: Methodology, Software, Supervision, Validation, Visualization, Writing – review and editing. AC: Conceptualization, Funding acquisition, Investigation, Methodology, Project administration, Resources, Supervision, Writing – original draft, Writing – review and editing.

## Footnotes

1
https://connectivity.brain-map.org

## Supplementary material

Supplementary Figure 1 Flow chart demonstrating the consecutive steps of the image analysis performed with the help of the Imaris system. CB, calbindin; CR, calretinin; TH, tyrosine hydroxylase.

Supplementary Video 1 Representative video sequence depicting a confocal laser microscope image stack from the NAc, doubly stained against TH and CR, loaded onto Imaris prior to quantitative analysis. Following manual rotation of the view angle, the TH+ axonal varicosity marked Juxt+ remains tightly adherent to a CR+ perikaryon, whereas the other TH+ bouton marked Juxt- appears to lose contact with the cell body, showing parallactic displacement of apparent position. Only the structure marked Juxt+ is considered to be a genuine close contact (juxtaposition).

## References

[B1] Abrahams B. S. Arking D. E. Campbell D. B. Mefford H. C. Morrow E. M. Weiss L. A. (2013). SFARI gene 2.0: A community-driven knowledgebase for the autism spectrum disorders (ASDs). *Mol. Autism* 4:36. 10.1186/2040-2392-4-36 24090431 PMC3851189

[B2] Ádám Á Kemecsei R. Company V. Murcia-Ramón R. Juarez I. Gerecsei L. (2020). Gestational exposure to sodium valproate disrupts fasciculation of the mesotelencephalic dopaminergic tract, with a selective reduction of dopaminergic output from the ventral tegmental area. *Front. Neuroanat.* 14:29. 10.3389/fnana.2020.00029 32581730 PMC7290005

[B3] Adiletta A. Pross A. Taricco N. Sgadò P. (2022). Embryonic valproate exposure alters mesencephalic dopaminergic neurons distribution and septal dopaminergic gene expression in domestic chicks. *Front. Integr. Neurosci.* 16:804881. 10.3389/fnint.2022.804881 35369647 PMC8966611

[B4] Alsanie W. F. Penna V. Schachner M. Thompson L. H. Parish C. L. (2017). Homophilic binding of the neural cell adhesion molecule CHL1 regulates development of ventral midbrain dopaminergic pathways. *Sci. Rep.* 7:9368. 10.1038/s41598-017-09599-y 28839197 PMC5570898

[B5] Antonopoulos J. Dori I. Dinopoulos A. Chiotelli M. Parnavelas J. G. (2002). Postnatal development of the dopaminergic system of the striatum in the rat. *Neuroscience* 110 245–256. 10.1016/S0306-4522(01)00575-9 11958867

[B6] Baronio D. Puttonen H. A. J. Sundvik M. Semenova S. Lehtonen E. Panula P. (2017). Embryonic exposure to valproic acid affects the histaminergic system and the social behavior of adult zebrafish (*Danio rerio*). *Br. J. Pharmacol.* 175 797–809. 10.1111/bph.14124 29235100 PMC5811620

[B7] Bayer S. A. Wills K. V. Triarhou L. C. Ghetti B. (1995). Time of neuron origin and gradients of neurogenesis in midbrain dopaminergic neurons in the mouse. *Exp. Brain Res.* 105 191–199. 10.1007/BF00240955 7498372

[B8] Bissonette G. B. Roesch M. R. (2016). Development and function of the midbrain dopamine system: What we know and what we need to. *Genes Brain Behav.* 15 62–73. 10.1111/gbb.12257 26548362 PMC5266527

[B9] Blaess S. Bodea G. O. Kabanova A. Chanet S. Mugniery E. Derouiche A. (2011). Temporal-spatial changes in Sonic Hedgehog expression and signaling reveal different potentials of ventral mesencephalic progenitors to populate distinct ventral midbrain nuclei. *Neural Dev.* 20:29. 10.1186/1749-8104-6-29 21689430 PMC3135491

[B10] Blakely B. D. Bye C. R. Fernando C. V. Horne M. K. Macheda M. L. Stacker S. A. (2011). Wnt5a regulates midbrain dopaminergic axon growth and guidance. *PLoS One* 6:e18373. 10.1371/journal.pone.0018373 21483795 PMC3069098

[B11] Borgkvist A. Puelles E. Carta M. Acampora D. Ang S. Wurst W. (2006). Altered dopaminergic innervation and amphetamine response in adult Otx2 conditional mutant mice. *Mol. Cell. Neurosci.* 31 293–302. 10.1016/j.mcn.2005.09.018 16256364

[B12] Bringas M. E. Carvajal-Flores F. N. López-Ramírez T. A. Atzori M. Flores G. (2013). Rearrangement of the dendritic morphology in limbic regions and altered exploratory behavior in a rat model of autism spectrum disorder. *Neuroscience* 241 170–187. 10.1016/j.neuroscience.2013.03.030 23535253

[B13] Cansler H. L. Wright K. N. Stetzik L. A. Wesson D. W. (2020). Neurochemical organization of the ventral striatum’s olfactory tubercle. *J. Neurochem.* 152 425–448. 10.1111/jnc.14919 31755104 PMC7042089

[B14] Chao O. Y. Pathak S. S. Zhang H. Dunaway N. Li J.-S. Mattern C. (2020). Altered dopaminergic pathways and therapeutic effects of intranasal dopamine in two distinct mouse models of autism. *Mol. Brain* 13:111. 10.1186/s13041-020-00649-7 32778145 PMC7418402

[B15] Chen J. Tian L. Lei L. Hou F. Roper C. Ge X. (2018). Development and behavior alterations in zebrafish embryonically exposed to valproic acid (VPA): Animal model of autism. *Neurotoxicol. Teratol.* 66 8–16. 10.1016/j.ntt.2018.01.002 29309833 PMC5856631

[B16] Cipriani A. Reid K. Young A. H. Macritchie K. Geddes J. (2013). Valproic acid, valproate and divalproex in the maintenance treatment of bipolar disorder. *Cochrane Database Syst. Rev.* 10 CD003196. 10.1002/14651858.CD003196 24132760 PMC6599863

[B17] Costes S. V. Daelemans D. Cho E. H. Dobbin Z. Parlakis G. Lockett S. (2004). Automatic and quantitative measurement of protein-protein colocalization in live cells. *Biophys. J.* 86 3993–4003. 10.1529/biophysj.103.038422 15189895 PMC1304300

[B18] Csillag A. Ádám Á Zachar G. (2022). Avian models for brain mechanisms underlying altered social behavior in autism. *Front. Physiol.* 13:1032046. 10.3389/fphys.2022.1032046 36388132 PMC9650632

[B19] Ergaz Z. Weinstein-Fudim L. Ornoy A. (2016). Genetic and non-genetic animal models for autism spectrum disorders (ASD). *Reprod. Toxicol.* 64 116–140. 10.1016/j.reprotox.2016.04.024 27142188

[B20] Fereshetyan K. Chavushyan V. Danielyan M. Yenkoyan K. (2021). Assessment of behavioral, morphological and electrophysiological changes in prenatal and postnatal valproate induced rat models of autism spectrum disorder. *Sci. Rep.* 11:23471. 10.1038/s41598-021-02994-6 34873263 PMC8648736

[B21] Finszter C. K. Kemecsei R. Zachar G. Holtkamp S. Echevarría D. Adorján I. (2023). Early cellular and synaptic changes in dopaminoceptive forebrain regions of juvenile mice following gestational exposure to valproate. *Front. Neuroanat.* 17:1235047. 10.3389/fnana.2023.1235047 37603782 PMC10435871

[B22] Fogarty M. J. Hammond L. A. Kanjhan R. Bellingham M. C. Noakes P. G. (2013). A method for the three-dimensional reconstruction of Neurobiotin™-filled neurons and the location of their synaptic inputs. *Front. Neural Circuits* 7:153. 10.3389/fncir.2013.00153 24101895 PMC3787200

[B23] Fuccillo M. V. (2016). Striatal Circuits as a Common Node for Autism Pathophysiology. *Front Neurosci.* 10:27. 10.3389/fnins.2016.00027 26903795 PMC4746330

[B24] Goodson J. L. (2005). The vertebrate social behavior network: Evolutionary themes and variations. *Horm. Behav.* 48 11–22. 10.1016/j.yhbeh.2005.02.003 15885690 PMC2570781

[B25] Göttlicher M. Minucci S. Zhu P. Krämer O. H. Schimpf A. Giavara S. (2001). Valproic acid defines a novel class of HDAC inhibitors inducing differentiation of transformed cells. *EMBO J.* 20 6969–6978. 10.1093/emboj/20.24.6969 11742974 PMC125788

[B26] Hamilton P. J. Campbell N. G. Sharma S. Erreger K. Herborg Hansen F. Saunders C. (2013). De novo mutation in the dopamine transporter gene associates dopamine dysfunction with autism spectrum disorder. *Mol. Psychiatry* 18 1315–1323. 10.1038/mp.2013.102 23979605 PMC4046646

[B27] Hara Y. Takuma K. Takano E. Katashiba K. Taruta A. Higashino K. (2015). Reduced prefrontal dopaminergic activity in valproic acid-treated mouse autism model. *Behav. Brain Res.* 289 39–47. 10.1016/j.bbr.2015.04.022 25907743

[B28] Hasue R. H. Shammah-Lagnado S. J. (2002). Origin of the dopaminergic innervation of the central extended amygdala and accumbens shell: A combined retrograde tracing and immunohistochemical study in the rat. *J. Comp. Neurol.* 454 15–33. 10.1002/cne.10420 12410615

[B29] Humphries M. D. Prescott T. J. (2010). The ventral basal ganglia, a selection mechanism at the crossroads of space, strategy, and reward. *Prog. Neurobiol.* 90 385–417. 10.1016/j.pneurobio.2009.11.003 19941931

[B30] Kataoka S. Takuma K. Hara Y. Maeda Y. Ago Y. Matsuda T. (2013). Autism-like behaviours with transient histone hyperacetylation in mice treated prenatally with valproic acid. *Int. J. Neuropsychopharmacol.* 16 91–103. 10.1017/S1461145711001714 22093185

[B31] Kim K. C. Kim P. Go H. S. Choi C. S. Yang S. I. Cheong J. H. (2011). The critical period of valproate exposure to induce autistic symptoms in Sprague-Dawley rats. *Toxicol. Lett.* 201 137–142. 10.1016/j.toxlet.2010.12.018 21195144

[B32] Klenowski P. M. Fogarty M. J. Belmer A. Noakes P. G. Bellingham M. C. Bartlett S. E. (2015). Structural and functional characterization of dendritic arbors and GABAergic synaptic inputs on interneurons and principal cells in the rat basolateral amygdala. *J. Neurophysiol.* 114 942–957. 10.1152/jn.00824.2014 26041829 PMC4725099

[B33] Kuo H.-Y. Liu F.-C. (2022). Pathophysiological studies of monoaminergic neurotransmission systems in valproic acid-induced model of autism spectrum disorder. *Biomedicines* 10:560. 10.3390/biomedicines10030560 35327362 PMC8945169

[B34] László K. Vörös D. Kiss O. László B. R. Ollmann T. Péczely L. (2022). The role of intraamygdaloid oxytocin and D2 dopamine receptors in reinforcement in the valproate-Induced autism rat model. *Biomedicines* 10:2309. 10.3390/biomedicines10092309 36140411 PMC9496370

[B35] Lauber E. Filice F. Schwaller B. (2016). Prenatal valproate exposure differentially affects parvalbumin-expressing neurons and related circuits in the cortex and striatum of mice. *Front. Mol. Neurosci.* 9:150. 10.3389/fnmol.2016.00150 28066177 PMC5174119

[B36] Liang C. L. Sinton C. M. German D. C. (1996). Midbrain dopaminergic neurons in the mouse: Co-localization with Calbindin-D28K and calretinin. *Neuroscience* 75 523–533. 10.1016/0306-4522(96)00228-X 8931015

[B37] Lorenzi E. Pross A. Rosa-Salva O. Versace E. Sgadò P. Vallortigara G. (2019). Embryonic exposure to valproic acid affects social predispositions for dynamic cues of animate motion in newly hatched chicks. *Front. Physiol.* 10:501. 10.3389/fphys.2019.00501 31114510 PMC6503819

[B38] Mahmood U. Ahn S. Yang E. Choi M. Kim H. Regan P. (2018). Dendritic spine anomalies and PTEN alterations in a mouse model of VPA-induced autism spectrum disorder. *Pharmacol. Res.* 128 110–121. 10.1016/j.phrs.2017.08.006 28823725

[B39] Maisterrena A. Matas E. Mirfendereski H. Balbous A. Marchand S. Jaber M. (2022). The state of the dopaminergic and glutamatergic systems in the valproic acid mouse model of autism spectrum disorder. *Biomolecules* 12:1691. 10.3390/biom12111691 36421705 PMC9688008

[B40] Martin L. J. Cork L. C. (2014). The non-human primate striatum undergoes marked prolonged remodeling during postnatal development. *Front. Cell. Neurosci.* 8:294. 10.3389/fncel.2014.00294 25294985 PMC4170103

[B41] Moldrich R. X. Leanage G. She D. Dolan-Evans E. Nelson M. Reza N. (2013). Inhibition of histone deacetylase in utero causes sociability deficits in postnatal mice. *Behav. Brain Res.* 257 253–264. 10.1016/j.bbr.2013.09.049 24103642

[B42] Mongia S. Yamaguchi T. Liu B. Zhang S. Wang H. Morales M. (2019). The ventral tegmental area has calbindin neurons with the capability to co-release glutamate and dopamine into the nucleus accumbens. *Eur. J. Neurosci.* 50 3968–3984. 10.1111/ejn.14493 31215698 PMC6920608

[B43] Nemoto C. Hida T. Arai R. (1999). Calretinin and calbindin-D28k in dopaminergic neurons of the rat midbrain: A triple-labeling immunohistochemical study. *Brain Res.* 846 129–136. 10.1016/s0006-8993(99)01950-2 10536220

[B44] Nguyen M. Roth A. Kyzar E. J. Poudel M. K. Wong K. Stewart (2014). Decoding the contribution of dopaminergic genes and pathways to autism spectrum disorder (ASD). *Neurochem. Int.* 66 15–26. 10.1016/j.neuint.2014.01.002 24412511

[B45] Nicolini C. Fahnestock M. (2018). The valproic acid-induced rodent model of autism. *Exp. Neurol.* 299 217–227. 10.1016/j.expneurol.2017.04.017 28472621

[B46] Nishigori H. Kagami K. Takahashi A. Tezuka Y. Sanbe A. Nishigori H. (2013). Impaired social behavior in chicks exposed to sodium valproate during the last week of embryogenesis. *Psychopharmacology (Berl)* 227 393–402. 10.1007/s00213-013-2979-y 23371491

[B47] O’Connell L. A. Hofmann H. A. (2011). The vertebrate mesolimbic reward system and social behavior network: A comparative synthesis. *J. Comp. Neurol.* 519 3599–3639. 10.1002/cne.22735 21800319

[B48] O’Connell L. A. Hofmann H. A. (2012). Evolution of a vertebrate social decision-making network. *Science* 336 1154–1157. 10.1126/science.1218889 22654056

[B49] Pavăl D. (2017). A dopamine hypothesis of autism spectrum disorder. *Dev. Neurosci.* 8 355–360. 10.1159/000478725 28750400

[B50] Pavăl D. Micluţia I. V. (2021). The dopamine hypothesis of autism spectrum disorder revisited: Current status and future prospects. *Dev. Neurosci.* 43 73–83. 10.1159/000515751 34010842

[B51] Paxinos G. Franklin K. B. J. (2001). *The mouse brain in stereotaxic coordinates: Hard cover edition.* Amsterdam: Elsevier.

[B52] Petryszyn S. Saidi L. Gagnon D. Parent A. Parent M. (2021). The density of calretinin striatal interneurons is decreased in 6-OHDA-lesioned mice. *Brain Struct. Funct.* 226 1879–1891. 10.1007/s00429-021-02298-5 34018041

[B53] Rodier P. M. Ingram J. L. Tisdale B. Nelson S. Romano J. (1996). Embryological origin for autism: Developmental anomalies of the cranial nerve motor nuclei. *J. Comp. Neurol.* 370 247–261. 10.1002/(SICI)1096-9861(19960624)370:2<247::AID-CNE8>3.0.CO;2-2 8808733

[B54] Rodríguez-López C. Clascá F. Prensa L. (2017). The mesoaccumbens pathway: A retrograde labeling and single-cell axon tracing analysis in the mouse. *Front. Neuroanat.* 11:25. 10.3389/fnana.2017.00025 28396627 PMC5367261

[B55] Roullet F. I. Lai J. K. Foster J. A. (2013). In utero exposure to valproic acid and autism–a current review of clinical and animal studies. *Neurotoxicol. Teratol.* 36 47–56. 10.1016/j.ntt.2013.01.004 23395807

[B56] Satterstrom F. K. Kosmicki J. A. Wang J. Breen M. S. De Rubeis S. An J. Y. (2020). Large-scale exome sequencing study implicates both developmental and functional changes in the neurobiology of autism. *Cell* 180 568–584. 10.1016/j.cell.2019.12.036 31981491 PMC7250485

[B57] Schätzle P. Wuttke R. Ziegler U. Sonderegger P. (2012). Automated quantification of synapses by fluorescence microscopy. *J. Neurosci. Meth.* 204 144–149. 10.1016/j.jneumeth.2011.11.010 22108140

[B58] Scherer S. W. Dawson G. (2011). Risk factors for autism: Translating genomic discoveries into diagnostics. *Hum. Genet.* 130 123–148. 10.1007/s00439-011-1037-2 21701786

[B59] Schiavi S. Iezzi D. Manduca A. Leone S. Melancia F. Carbone C. (2019). Reward-related behavioral, neurochemical and electrophysiological changes in a rat model of autism based on prenatal exposure to valproic acid. *Front. Cell. Neurosci.* 13:479. 10.3389/fncel.2019.00479 31708750 PMC6824319

[B60] Schneider T. Przewlocki R. (2005). Behavioral alterations in rats prenatally exposed to valproic acid: Animal models of autism. *Neuropsychopharmacology* 30 80–89. 10.1038/sj.npp.1300518 15238991

[B61] Sgadò P. Rosa-Salva O. Versace E. Vallortigara G. (2018). Embryonic exposure to valproic acid impairs social predispositions of newly hatched chicks. *Sci. Rep.* 8:5919. 10.1038/s41598-018-24202-8 29650996 PMC5897402

[B62] Snyder-Keller A. Tseng K. Y. Lyng G. D. Graber D. J. O’Donnell P. (2008). Afferent influences on striatal development in organotypic cocultures. *Synapse* 62 487–500. 10.1002/syn.20518 18435420

[B63] Staal W. G. de Krom M. de Jonge M. V. (2012). Brief report: The dopamine-3-receptor gene (DRD3) is associated with specific repetitive behavior in autism spectrum disorder (ASD). *J. Autism Dev. Disord.* 42 885–888. 10.1007/s10803-011-1312-z 21691864 PMC3324694

[B64] Supekar K. Kochalka J. Schaer M. Wakeman H. Qin S. Padmanabhan A. (2018). Deficits in mesolimbic reward pathway underlie social interaction impairments in children with autism. *Brain* 141 2795–2805. 10.1093/brain/awy191 30016410 PMC6113649

[B65] Voorn P. Kalsbeek A. Jorritsma-Byham B. Groenewegen H. J. (1988). The pre- and postnatal development of the dopaminergic cell groups in the ventral mesencephalon and the dopaminergic innervation of the striatum of the rat. *Neuroscience* 25 857–887. 10.1016/0306-4522(88)90041-3 3405431

[B66] Wagner G. C. Reuhl K. R. Cheh M. McRae P. Halladay A. K. (2006). A new neurobehavioral model of autism in mice: Pre- and postnatal exposure to sodium valproate. *J. Autism Dev. Disord.* 36 779–793. 10.1007/s10803-006-0117-y 16609825

[B67] Wu H. F. Chen P. S. Chen Y. J. Lee C. W. Chen I. T. Lin H. C. (2017). Alleviation of N-methyl-D-aspartate receptor-dependent long-term depression via regulation of the glycogen synthase kinase-3β pathway in the amygdala of a valproic acid-induced animal model of autism. *Mol. Neurobiol.* 54 5264–5276. 10.1007/s12035-016-0074-1 27578017

[B68] Yamaguchi H. Hara H. Ago Y. Takano E. Hasebe S. Nakazawa T. (2017). Environmental enrichment attenuates behavioral abnormalities in valproic acid-exposed autism model mice. *Behav. Brain Res.* 333 67–73. 10.1016/j.bbr.2017.06.035 28655565

[B69] Yu Z. Guindani M. Grieco S. F. Chen L. Holmes T. C. Xu X. (2022). Beyond t test and ANOVA: Applications of mixed-effects models for more rigorous statistical analysis in neuroscience research. *Neuron* 110 21–35. 10.1016/j.neuron.2021.10.030 34784504 PMC8763600

[B70] Zachar G. Tóth A. S. Gerecsei L. I Zsebők S. Ádám Á Csillag A. (2019). Valproate exposure in ovo attenuates the acquisition of social preferences of young post-hatch domestic chicks. *Front. Physiol.* 10:881. 10.3389/fphys.2019.00881 31379596 PMC6646517

