# Supplementary figures and images for: Gestational VPA exposure reduces the density of juxtapositions between TH+ axons and calretinin or calbindin expressing cells in the ventrobasal forebrain of neonatal mice
Source: Front Neuroanat. 2024 Jul 4;18:1426042. doi: 10.3389/fnana.2024.1426042 (PMC11254666; doi:10.3389/fnana.2024.1426042)

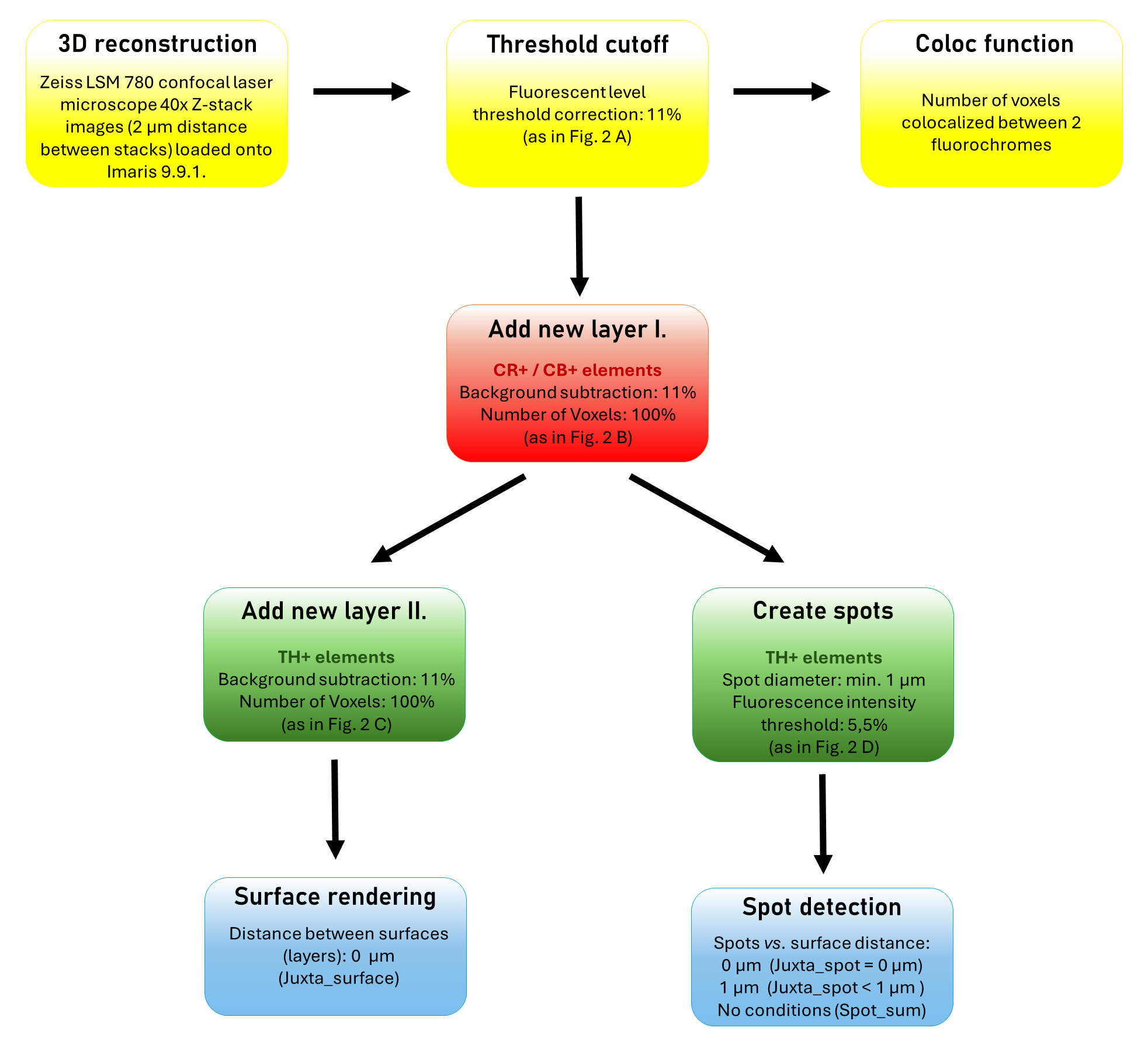

Supplement: Supplementary Figure 1 — Flow chart demonstrating the consecutive steps of the image analysis performed with the help of the Imaris system. CB, calbindin; CR, calretinin; TH, tyrosine hydroxylase. [file Image_1.TIF]
